# Shaping the abortion policy – competing discourses on the Zambian termination of pregnancy act

**DOI:** 10.1186/s12939-018-0908-8

**Published:** 2019-01-28

**Authors:** Marte E. S. Haaland, Haldis Haukanes, Joseph Mumba Zulu, Karen Marie Moland, Charles Michelo, Margarate Nzala Munakampe, Astrid Blystad

**Affiliations:** 1 Centre for international Health, Department of Global Public Health and Primary Care, University of Bergen, Bergen, Norway; 2 Centre for Intervention Science in Maternal and Child Health (CISMAC), University of Bergen, Bergen, Norway; 3 Department of Health Promotion and Development, University of Bergen, Bergen, Norway; 4 School of Public Health, University of Zambia, Lusaka, Zambia

**Keywords:** Sexual and reproductive health and rights, Abortion, Health policy

## Abstract

**Introduction:**

The Zambian Termination of Pregnancy Act permits abortion on socio-economic grounds, but access to safe abortion services is limited and this constitutes a considerable problem for rights to sexual and reproductive health. The case of Zambia provides an opportunity to explore the relationship between a legal framework that permits abortion on diverse grounds, the moral and political disputes around abortion and access to sexual and reproductive health services.

**Methods:**

This paper draws upon eleven months of ethnographic fieldwork in Zambia. The fieldwork included 28 open-ended interviews with key stakeholders as well as the collection of archival material related to the origins of Zambia’s legal framework for abortion. The archival material and the interview data were analyzed thematically, using theoretical perspectives on discourse and the anthropology of policies.

**Results:**

The study findings show that the Zambian case is not easily placed into standard categories of liberal or restrictive abortion laws. The archival material reveals that restrictive elements were in focus when the Zambian Termination of Pregnancy Act was passed (1972). The restrictive aspects of the law were emphasized further when Zambia was later declared as a Christian nation. Some of these restrictive elements are still readily recognized in today’s abortion debate. Currently there are multiple opinions on whether Zambian abortion policy is liberal, restrictive or neither. The law emerges as ambiguous, and this ambiguity is actively used by both those working to increase access to safe and legal abortion services, and those who work to limit such access. Coupled with a lack of knowledge about the law, its ambiguity may work to reduce access to safe abortion services on the grounds permitted by the law.

**Conclusions:**

We argue that the Zambian Termination of Pregnancy Act is ambiguous and leaves much room for interpretation. This paper challenges the notion that the Zambian abortion law is liberal and opens up for further discussion on the relationship between how a law is described and perceived by the public, and the rights to health and services ensured by it.

## Introduction

Since the Termination of Pregnancy Act was passed in 1972 [1], Zambia has been seen internationally as having a liberal legal framework for abortion [2, 3]. Less restrictive legal conditions are often coupled with lower estimates of illegal and unsafe abortions [4], but in Zambia, where complications from unsafe abortions are a considerable problem [5, 6], this does not seem to be the case. This apparent paradox allows us to explore the entangled relationship between the existence of a legal framework that permits abortion, the moral and political disputes associated with the topic of abortion, and the right to access sexual and reproductive health services.

When the Termination of Pregnancy Bill was proposed to the Zambian parliament in 1972, it was drafted on the basis of the United Kingdom Abortion Act of 1967 [1, 7]. The Zambian Act that was approved by parliament allows abortion to protect the physical or mental health of the pregnant woman or any of her existing children. It also allows abortion in the case of serious fetal malformation [1]. The law specifies that the pregnant woman’s age and environment should be taken into consideration [1] and therefore allows for legal abortions on broad socio-economic grounds. Simultaneously, the law spells out some restrictions for access to legal abortion services. It specifies that abortions must only be provided in registered hospitals, and that three medical doctors - one of whom must be a specialist in the field in which the grounds for the legal abortion is being sought - must sign the form to allow the procedure to take place [1]. In cases of medical emergency, the signature of one medical doctor is sufficient. The law moreover permits conscientious objection, allowing health practitioners to abstain from providing abortion services on religious grounds, except in emergency situations [1]. Abortions are also regulated by the criminal code. The code establishes penalties of up to 7 years for those who illegally provide abortion services, and up to 14 years for women who procure illegal abortions or anyone who assists her [8]. It is only abortions that follow the procedures of the Termination of Pregnancy Act that are not criminalized. In 2005, the criminal code was amended to ensure that girls who are victims of rape can seek legal termination of pregnancy [8]. Zambia has ratified the Maputo protocol committing to provide comprehensive reproductive health services, including safe abortion [9]. Despite these legal commitments, abortion remains a controversial issue in Zambia. In 2015, a clause stating that “the right to life begins at conception”, was included in a proposed new Bill of Rights of the Zambian constitution, implying a legal move in a more restrictive direction [10]. In August 2016, the Bill of Rights was sent to a referendum that was declared unsuccessful due to low turnout [11], but, with the proposition, abortion re-emerged on the Zambian political agenda as a contentious issue. In Zambia, religion and politics have been closely intertwined since independence and church bodies such as the Catholic Church (represented by the Zambian Episcopal Conference) and the protestant churches have had a great deal of political influence [12]. During the last decades, Pentecostal churches have also become more influential, and with the declaration of Zambia as a Christian Nation in 1991, the links between religion and politics became even more explicit [13]. This paper addresses the consequences this has had for the way the Zambian abortion policy has been contested and disputed since its introduction.

Zambian abortion policy cannot be understood separately from the structural conditions of the Zambian health system that is facing a crisis of human resources [14]. In 2016, there were only 1514 employed medical doctors in the country [14] for a population of 16.2 million [15]. This places Zambia among the 25 African Countries with less than 1 doctor per 10,000 inhabitants [16]. More than 60% of the population lives in rural areas where lack of qualified health personnel is particularly acute and facilities are often understaffed [14]. A newly revised set of standards and guidelines for comprehensive abortion care specifies that an abortion can be carried out with the signature of only one medical doctor if no other are available [17]. However, this new regulation has not been disseminated to health care workers across the country. Though mid-level providers such as midwives, nurses or clinical officers can carry out the abortion procedures, the law specifically requests the approval signatures from medical doctors. Lack of medical doctors, thus, continues to constitute an important barrier to accessing legal abortion services, that is unevenly distributed across the country.

In our examination of the history and current perceptions of Zambian abortion policy, we draw upon the work of Shore and Wright [18, 19] and their ‘anthropology of policies’. Policies are manifested in many different ways, including, laws, regulations, and actions of state officials or civil servants. In this paper, we approach Zambian abortion policy through the Termination of Pregnancy Act. Paraphrasing Appadurai [20], Shore and Wright highlight the social ‘lives’ of policies [21] and point out how policies may appear as fixed, but are in fact, continuously contested and reshaped. As the context of a policy changes with time or space, its meaning also changes in ways that have consequences beyond its original intentions [21]. This perspective allows us to examine how diverging images of and narratives about the abortion policy travel through time and settings, and continuously reshape ideas of what a legal abortion is. The Foucauldian concept of discourse as a way of describing the complex relationship between power, truth and right [22] is also central to the analysis. Discourse sets limits to how a topic can be addressed or conceived in a meaningful way and “the power embedded in discourse will determine what people consider to be true” [23]. These perspectives allow us to analyse how images created as notions of ‘truths’ about abortion policy have powerful implications for the range of actions available to girls and women making reproductive choices.

There is a general lack of data on the prevalence and characteristics of both legal and illegal abortions in Zambia [24]. The last official estimate from the Zambian authorities in 2017 asserts that 30–50% of all acute gynaecological admissions are abortion related and that six per thousand women die of abortion complications, most of them caused by unsafe abortions [17]. There are no official data on number and circumstances of legal abortions. Studies have found that, in Zambia, both knowledge about the legal status of abortion and access to abortion services are poor [2, 3, 24, 25]. To the best of our knowledge, however, no study has explored the history of abortion policy and how this history is entangled in current policy debates about abortion. This paper, therefore, examines historical, political and discursive elements of Zambian abortion policy and offers a new perspective on the legal framework for abortion in Zambia. The paper traces the moral and political context of 1972 to today and argues that Zambia’s abortion law is inherently ambiguous and allows continuous contestations that shape abortion policy in ways that influence girls’ and women’s access to sexual and reproductive health services. In the process, the paper brings into question the international view of Zambian abortion law as being liberal.

## Methods

This study is part of a multi-sited ethnographic study on the sociopolitical dimensions of access to fertility control and safe abortion in Zambia. It forms part of the comparative research project “Competing discourses impacting girls’ and women’s rights: Fertility control and safe abortion in Ethiopia, Zambia and Tanzania” funded by the Norwegian Research Council and the University of Bergen, Norway [26]. The umbrella project aims to explore the articulation between legal abortion frameworks, public discourses on abortion, and access to fertility control and safe abortion services in three African countries with different legal frameworks for abortion.

The present paper draws upon ethnographic fieldwork in Zambia between September 2017 and August 2018, which included the collection of media coverage on issues of sexual and reproductive health, collection of archival material, open-ended interviews with key actors as well as informal conversations with relevant stakeholders. Though informed by all of these sources, the findings presented in this paper are primarily based on the archival material and the open-ended interviews.

In October 2017, we accessed the Zambian National Archives and consulted the private collection of the Speaker of the Zambian parliament in 1972, Robinson Nabulyato. The collection included his correspondence with the first Zambian president Kenneth Kaunda on the Termination of Pregnancy Act. The library of the Zambian National Assembly was accessed in December 2017 to collect records of the parliamentary debates about the Termination of Pregnancy Bill in 1972, as well as the parliamentary debates about the amendment of the Penal Code in 2005. All the collected archival material was copied into text documents for analysis. To complement the archival material, we carried out an open-ended interview with a person employed as a high-ranking official in the Ministry of Health in 1972. The interviewee had participated in the preparation of the Termination of Pregnancy Bill and in the parliamentary process leading to its enactment.

We conducted 28 open-ended interviews with stakeholders who are influential in shaping Zambia’s abortion policy or public opinion on reproductive health issues. The interviews covered topics about the interviewees’ views about the abortion law and policy both today and historically, as well as their engagement and advocacy work with regards to sexual and reproductive health and rights in Zambia. Some interviews were conducted with two or more interviewees. When selecting potential interviewees, a list was made of ministries, NGOs (national and international), international agencies, religious bodies and professional health sector organizations who had been influential in abortion-related debates or that have political influence in other ways. We used snowball sampling to expand the list, and made efforts to secure interviews with a broad range of actors aiming to include different points of view [27]. The large majority of the stakeholders we approached for interviews accepted the invitation, but some declined arguing that the topic was not relevant to their area of work. A few never responded to our request. All interviews were conducted in English, the official language in Zambia, and were recorded with the informants’ consent and transcribed verbatim. Most interviewees spoke at length about their views and opinions about Zambia’s abortion policy giving examples from their own experience with the matter.

We analyzed the collected material through a critical and reflexive lens using a thematic approach. Both the interviews and the archival material were carefully read and re-read before they were imported into Nvivo11, which was used as a tool for inductively coding and clustering into categories of both types of data. Based on rounds of careful review and categorizing, three pronounced themes were selected for further analysis. These were: the distinct focus on the restrictive elements of the law in 1972; the changing historical context of the law; and the current conflicting perceptions of the law. Shore and Wright’s theoretical framework on policy [18, 21] was used to guide the analysis and writing processes. The quotes presented in the findings section to represent the major themes, derive from both the archival material and the interviews.

The paper could have been strengthened by findings from participant observation of the daily activities of one or more of the actors central in influencing the Zambian abortion policy. Due to barriers of access, this method was not possible to include in this part of the ethnographic study. Future papers from the same study will include findings from participant observation among regional and local level policy makers and daily life in communities of Zambia’s Western Province. Nevertheless, the archival material and the interviews this paper builds upon are well suited for exploring the historical, political and discursive elements of the Zambian abortion policy that this paper addresses.

The University of Zambia Biomedical Research Ethics Committee (009–07-17) and the Regional Ethical Committee Western Norway (2017/1191), granted ethical approval for this study. All participants gave informed consent.

## Findings

### Making the termination of pregnancy act

The Zambian Termination of Pregnancy Act was passed in 1972, 8 years after Kenneth Kaunda became the first president of the independent Republic of Zambia in a time-period when the country was transitioning into a one party system [28]. President Kaunda’s nation-building ideological project was named ‘Zambian Humanism’ and has been described as a mix of socialism, liberalism, Christian morality and idealized communal values of the pre-colonial past [29]. Whether Zambian Humanism was translated into politics and action has been questioned [29:160]. For the purpose of this paper, the ideological project provides insight into how the political elite at the time wished, ideologically and morally, to shape the interactions between the post-colonial Zambian state and its citizens [30, 31]. Zambian Humanism encompassed a conservative Christian morality combined with patriarchal perspectives on the female body and sexuality. The ruling party UNIP published a moral code in 1975 expressing conservative views on issues such as the length of women’s skirts and women’s limited rights over their own sexuality and body [31]. Against this contextual backdrop, we now turn to the debates leading up to the parliament’s adoption of the Zambian Termination of Pregnancy Act.

Before the Termination of Pregnancy Bill became an act in 1972, the practice of abortion was regulated by the Penal Code that criminalized both the abortion-seeking woman, any of her helpers, and the abortion provider. The records from the parliamentary debate indicate that clandestine abortions were common in 1972. Nevertheless, addressing this phenomenon by increasing access to safe and legal abortion services was not explicitly on the agenda when the Bill was proposed. The Minister of Health who presented the Termination of Pregnancy Bill to the Members of Parliament rather argued that it was proposed as a simple clarification of the existing legal framework for abortion:

*Mr Speaker, Sir, the purpose of this Bill is to amend and clarify the law relating to termination of pregnancy by registered medical practitioners. The bill provides for a stricter control of termination of pregnancy in that it requires two registered medical practitioners and a specialist in the branch of medicine in which the patient is specifically required to be examined before a conclusion is reached that the abortion should be recommended* (Zambian Parliamentary Library, Parliamentary Debates 1. August 1972).

The aim of clarifying the legal framework for abortion was repeated by the Minister throughout the parliamentary session. He also used public health arguments when presenting the Bill, arguing that the new law would remove doubts among medical practitioners about when abortions were indeed legal and therefore prevent unnecessary deaths from sepsis and bleeding caused by some unsafe clandestine abortions (Zambian Parliamentary Library, Parliamentary Debates 1. August 1972).

The Bill received some opposition during the debate, but it finally passed by 66 to 13 votes. The Members of Parliament who opposed the Bill used a range of different arguments, including that the new law would impede desired population growth and that it opposed Zambian cultural norms condemning abortion. Some also appealed to moral arguments either about the abortion itself or about undesired aspects of women’s sexuality they believed the Bill would promote. A few argued that the law text would condone and increase the overall number of abortions. Nevertheless, the ideas and values expressed by most of the opposing Members of Parliament were not radically different from those expressed by the Minister of Health, as can be seen in the following statements by one of the opponents to the Bill:

*I am not one of those extremists, Mr Speaker, Sir, who would add that under no circumstances should abortion ( … ) be authorised. That would be an extreme case, and if this was the situation in this country that abortion was not allowed under whatever circumstances, I would have welcomed the Bill myself (*Zambian Parliamentary Library, Parliamentary Debates 1. August 1972)*.*

This statement expresses a position on abortion as something to be restricted and strictly regulated, while also allowing it in a few and very exceptional cases. The disagreement between the supporters and opponents of the law was not mainly a discussion for or against women’s general access to abortion services, but rather about the need for a specific law for abortion to allow it in such very specific situations. The Minister concluded the parliamentary debate by repeating the emphasis on the restrictive nature of the law stressing that it does “*not open the flood-gates for termination of pregnancy upon demand. There is nothing in the Bill which says so” (*Zambian Parliamentary Library, Parliamentary Debates 1. August 1972).

### Reasons and reactions

After the Termination of Pregnancy Bill was approved in parliament and before President Kaunda gave his assent, turning it into law, the Catholic Church in Zambia protested against the Bill in a letter addressed to the Secretary General to the Cabinet (Letter from Secretary General of the Zambian Episcopal Conference to Secretary General to the Cabinet, 12.8.1972, Zambia’s National Archives, Robinson Nabolyato’s collection HM/79/PP/1/72/5). The letter used a variety of arguments to oppose the Bill, including criticism of its quick processing in parliament, the lack of public debate about the topic and the adoption of a UK law in a recently independent Zambia. The protest letter was discussed in personal correspondence between the Speaker of the Parliament and the President, casting further light on the intentions behind the law. The Speaker emphasized that the law was introduced to clarify an existing confusion about the abortion legislation provided in the penal code, adding to the President that*: “For somebody to think that it is a new law for terminating pregnancies on a wholesale basis is most irresponsible”* (Letter from Speaker Robinson Nabolyato to President Kenneth Kaunda, 24.8.1972, Zambia’s National Archives, Robinson Nabolyato’s collection HM/79/PP/1/72/5)*.* He further stressed that the law was not intended to make abortion services available on request, a point that was made clearer when he wrote about the political consequences if the President should not approve the new legislative act with his assent. This, he argued, would allow the Catholic Church to influence decisions already made in Parliament. He claimed that:

*Such action would be a highly expensive exercise involving and disturning the whole country while the Bill itself is meant to serve two to five people in twenty years or so.*

The archival documents paint a picture of a process where the Termination of Pregnancy Act was introduced to clarify the legal status of abortion, not to liberalize it. Why the lawmakers chose to include socio-economic grounds for abortion remains unclear in the documents. An interview with a retired high-ranking official of the Ministry of Health involved in preparing the Bill in 1972 provided further insights into the law-making process. He expressed that the act was made:

*( … ) just to allow genuine cases, like say for instance where a mother has German measles or there is a risk to her mental health. But it is in our country, very limited circumstances under which a pregnancy would be terminated.*

In line with what we found in the parliamentary debates, the former official argued that the main purpose of the law was for doctors to be able to perform abortions legally on medical grounds. He did not mention more directly socio-economic cases such as poverty among adult women. When asked about the rationale for introducing an abortion law that allows abortion beyond strictly medical grounds, he said that there were a few non-medical cases they had in mind, such as girl children who were victims of rape. This is an interesting observation since the Termination of Pregnancy Act makes no reference to rape. Only in 2005 was the penal code amended to allow abortions for rape victims.

Rather than aiming for liberalization, the retired policy maker referred to how they, in 1972, were concerned with restricting the practice of abortion. That is the reason he gave for including the requirement for three signatures from medical practitioners including a specialist. Reflecting on this measure, he stated: “*These things are very difficult to be precise about. To balance between* [what is] *fair, liberal, reasonable and too restrictive.”* In retrospect, he seemed to be of the opinion that the wording of the Act may have been too permissive:

*It was a progressive move, and even with the restrictions in the Termination of Pregnancy Act, there was the likelihood that there could be abuses. So in future, maybe we need to tighten the restrictions.*

### Abortion in a Christian nation

When Zambia’s second president, Fredrick Chiluba, who identified as a born-again Christian, was elected in 1991, he brought a set of Pentecostal symbols and rhetoric to the state house [13, 32]. Though the major churches and Christian morality had been influential during Kaunda’s presidency from 1964 to 1991 [12, 31, 33], Christianity took a more explicit role in the 1990s that further influenced the relationship between citizens and the state. Only a few months into his presidency, Chiluba declared Zambia a Christian Nation, and in 1996 this declaration was included in the preamble to the national constitution. Much has been written about how the declaration of Zambia as a Christian Nation came about and what reactions it caused [13, 30, 32–35]. Particularly relevant to this paper is how the declaration came to shape dominant discourses on morality, sexuality and reproduction. Van Klinken [31] demonstrates how the declaration of Zambia as a Christian Nation is often used as a moral argument in public debates on issues such as pornography, sex work, women’s dress codes and gay rights, but the declaration also affects the politics of abortion in Zambia.

An employee and activist in an NGO working with sexual and reproductive health and rights described the entangled relationship between morality, religion and politics in Zambia in the following manner:

*I am sure you have already heard a thousand times that Zambia is a Christian Nation, so we* [Zambians] *tend to use that to not talk about what is considered taboo topics. ( … ) In terms of abortion, the moralistic view usually comes into play, and so whenever there is a conversation around abortion there is always a moralistic and religious argument that comes.*

The connection made between the Christian Nation and “moralistic religious arguments” is common. By referring to the Christian Nation, a person could readily take on a moral high-ground, and simultaneously silence diverging views on issues relating to sexual and reproductive morals, including abortion.

The discursive power of the ‘Christian Nation’ idiom goes beyond its use as an argument in debates. It also influences which laws and policies are conceivable and politically possible. On the question on whether the Termination of Pregnancy Act would have passed, had it been presented to parliament today rather than in 1972, several of the interviewees referred to the declaration as an impeding factor. One of the journalists interviewed phrased the point as follows:

*I think the reason why it was easy to pass such a law in 1972 was because Zambia had not been declared a Christian Nation yet. But, from 1991 when this country was declared a Christian Nation, there has been this complication when it comes to such issues* [abortion]*, because people feel we are a Christian Nation and we have to live by the Christian values.*

This statement shows the wide-ranging discursive and disciplining effects of the declaration of Zambia as a Christian nation. The Christian Nation idiom is tied to individuals’ morality and way of life. It has been noted that speaking of politics as a question of personal morality is characteristic for the vernacular of politics in Zambia, and the use of the Christian Nation idiom seems to be particularly useful in creating such a nexus between person and politics in a way that shapes both public debates and political possibilities [36, 37].

The relationship between the Christian Nation and the actions of its citizens emerges clearly when discussing health practitioners’ use of conscientious objection to abstain from taking part in terminations of pregnancies. As an Obstetrics and Gynaecology specialist in a private hospital explained:

*There is also the aspect of the medical practitioners who are willing to do the legal termination* [of pregnancy]. *So, being a Christian Nation and being declared as such, very few would want to be identified as one who does legal termination* [of pregnancies]*.*

An employee of an NGO and abortion rights advocate recalls how the situation at the gynaecological wards changed during her years working as a young nurse when President Chiluba came to power:

*He declared Zambia as a Christian Nation and now everybody was saying ‘why should we be terminating pregnancy when we are a Christian Nation?’ ( … ) Then the doctors asked themselves ‘Am I Christian? Should I continue?’ So they would withdraw and say ‘No, not my area’, but before it was politicized, they* [the doctors] *were signing* [forms authorizing abortions]*.*

These statements show a connection between the declaration of Zambia as a Christian Nation and access to safe and legal abortion services. They underline the fact that the declaration may have influenced the willingness and ability of doctors to provide the necessary signatures for a legal abortion to be carried out. In this way, access to safe and legal abortion services was further restricted.

### Talking about the law

Many of the interviewed stakeholders used the labels ‘liberal’ or ‘restrictive’ when characterizing the law. Interpreting the law in a way that would make it possible to grant legal and safe abortion services to a broad spectrum of abortion-seeking women emerged as an important reason for representing the law as liberal. The employee of an NGO working with sexual and reproductive health and rights described how the grounds for legal abortion listed in the law leave room for interpretation and access to safe abortion as follows:

*It* [the law] *is open to broader interpretation, ( … ) If I* [as an abortion-seeking woman] *explain my circumstances, you will see how they mostly connect to either one of those provisions* [allowing abortions]*, so it* [the law] *is really broad and opens up for women’s access.*

In other instances, the law was described as liberal in order to highlight the discrepancy between the broad grounds for legal abortions and the few women accessing safe and legal services. In the context of the number of illegal and unsafe termination of pregnancies, an advocate and provider of legal abortions told us the following:

*Nobody should die from unsafe abortion and nobody should have an unsafe abortion in the environment where the law is very liberal*.

Other stakeholders would, in contrast, use the term ‘restrictive’ when characterizing the law. Some emphasized what they called the ‘medical’ aspects of the law, referring to the terminology of physical and mental health in the law and the requirement of three signatures from medical practitioners. A high-ranking representative from a religious organization commented upon the Termination of Pregnancy Act saying:

*It is restrictive, and it has more to do with therapeutic* [abortions]. *Like in the case of losing a mother, the woman who is pregnant. It’s not given like a norm, - it is for the exceptions*.

The emphasis on the medical aspects of the law was repeated by other actors, including national level bureaucrats and health professionals who based this view on the requirement for three medical signatures. A national level bureaucrat emphasized the necessity of doctors’ signatures on a certificate for a legal abortion to take place when saying: “*I will start by saying that in Zambia abortion is illegal except when there is a certificate from a medical person.”*

Interestingly, the grounds on which abortion is allowed seem to be used as a basis of the argument for both those stakeholders arguing that the law is liberal and for those arguing that it is restrictive. The law thus seems to be ambiguous enough to allow for two very different positions. The ambiguity of the law creates a somewhat confusing scenario. An NGO employee and advocate tried to explain people’s confusion about the legal status of abortion in Zambia in the following terms:

*They will tell you it is illegal because it has all these requirements* [for when an abortion is legal], *and if it is legal then it should not have requirements. But, I think that is the understanding and interpretation*. *That’s partly why people are confused.*

The confusion surrounding the law was also expressed by a retired women’s rights activist:

*It was much later that I was actually fighting for a legal abortion law. Someone told me, ‘you already have it’, but at that point I didn’t look at it that way. All I could see were so many don’ts and doctors and I said that is not good enough. ( … ) And there was lot of dependence on medical, - it had to be medical reasons.*

Some stakeholders argued that the Termination of Pregnancy Act was neither liberal nor restrictive, but that it rather strikes a balance between granting access to the services, without making it what they considered to be ‘too widely accessible’. Answering a question on whether the Termination of Pregnancy Act would have been passed had it been presented today, a representative from a professional association answered:

*I think it would have passed. Because it is not too restrictive and again it is not too liberal ( … ) Because I think any law should be able to provide checks and balances, if you make it very liberal the street corner* [practitioner] *can have an abortion service and I think it may not be best for the country.*

While not all stakeholders would use terms like ‘liberal’ and ‘restrictive’ to characterize the law, many argued that the law did not grant women access to safe and legal abortion services. Some described the law itself as the greatest impediment to access. A national level policy maker with expertise in maternal health described the challenge in the following manner:

*I think the major obstacle today is the law because it does not provide abortion. You know there are all these requirements but when you look at the law itself it is not yet clear to say that actually a girl who falls pregnant can get access to the facility and be able to access safe abortion. The law is there, but accessibility and utilization are still limited.*

## Discussion

Our findings reveal that the Zambian Termination of Pregnancy Act is an ambiguous law that leaves much room for interpretation. Though the law in 1972 appeared to be progressive, allowing abortions on socio-economic grounds, the archival material reveals that the restrictive elements of the law were in focus in the debates preceding its enactment. These same elements were further strengthened with the declaration of Zambia as a Christian Nation that had important consequences for health workers’ will to be involved in legal abortion services. The restrictive elements of the law can readily be recognized in today’s abortion debate in which the Zambian abortion policy is continuously contested.

In international fora, the notion that Zambia has a liberal abortion policy is widespread. The findings from our study reveal that there is no consensus about this representation of the law within Zambia itself. Global actors involved in analyzing or advocating for abortion rights worldwide are part of the process of presenting this image of Zambian abortion law. Researchers [38, 39], and international NGOs [40] have compiled and compared abortion-related legislation from different countries on several occasions and in such comparisons, Zambia is presented as having a liberal or permissive legal framework for abortions. However, when legal frameworks are compared, they are necessarily simplified and decontextualized. Commonly, comparisons of abortion laws are presented as tables where the grounds under which legal abortions are allowed are listed [38–40]. In such comparisons, the Zambian law will appear as liberal, as it permits legal abortions for more indications than most countries in Sub-Saharan Africa. However, this only paints part of the picture. The comparisons rarely take into account components of the legal framework that may have strongly restrictive implications, such as the requirement for signatures from two medical doctors and a medical specialist, or that legal abortions can only be carried out in registered hospitals [1]. By presenting the law in a decontextualized manner, and focusing only on one part of it, a partial picture that supports the image of a liberal abortion law in Zambia is allowed to prevail.

The history of the law presented in this paper tells a more nuanced story. The law text itself seemed progressive for its time as it opened up for legal abortion on more than only medical grounds. However, the archival material reveals that the restrictive elements of the law were just as important parts of the conceptualization when introducing and defending The Termination of Pregnancy Bill. The inherent contradiction of the law, allowing abortions on socio-economic grounds on the one hand, while seriously restricting access to abortion services on the other, can be understood as an attempt to balance the liberal aspects of the law with restrictions. Though the wording of the Termination of Pregnancy Act is very similar to the British Abortion Act of 1967, one important difference should be noted. Where the British law requires the approval of two medical practitioners for an abortion to be legal, the Zambian law requires the additional signature of a specialist within the field of medicine relevant to the ground under which abortion is requested. Considering the low ratio of medical specialists to inhabitants in Zambia, this constitutes a major restriction to accessing legal abortion [41] .

Examining the discourse surrounding how the Termination of Pregnancy Act became law enhances our understanding of how the law is perceived and practiced today. Shore and Wright argue that the passing of a law is a special moment in a continuous process of contestation, when one point of view succeeds in “making their versions authoritative and embedding it in the precepts and procedures of the state” [21] and effectively silencing others. Following this line of thought, the debates surrounding the Termination of Pregnancy Bill provide insight into what opinions and values became the dominant authoritative discourses on abortion in Zambia. When we scrutinize the views that were expressed by the advocates for the law in 1972, we find that they were indeed located fairly closely to the views of their opponents; both sides were arguing for a clear law that would regulate and restrict access to abortion services. Whether the supporters of the act strategically employed a discourse of restriction in order to increase the chances that the law would be approved remains unknown. The Speaker’s estimation of cases eligible for legal abortion to ‘between two and five in twenty years’, seems so far from reality that it raises questions. Nevertheless, regardless of the policy makers’ agenda, the imagery of a liberal abortion law intended to expand access to safe abortion services was not used and made authoritative during the process of enacting the abortion law. Instead, more conservative and restrictive imagery was used in which access to legal abortion was to be strictly regulated. This resonates with the dominating moral discourse of the time (1970s), which was characterized by conservative and patriarchal views on female sexuality and where special moral scrutiny was applied to the female body by the state as well as by the general public [31].

### Who labels the law?

This study has revealed diverging opinions on whether the Zambian law is liberal or restrictive. The actors working to expand access to legal abortion services often used the term ‘liberal’ to highlight the contrast between a legal framework allowing abortions and girls and women’s access to abortion services. Though they seldom would express their argument as a question of rights, this emerged to be an underlying reasoning. Since the International Conference on Population and Development in Cairo, 1994, and the World Conference on Women in Beijing, 1995, abortion has been framed as a sexual and reproductive right and thereby an integral part of human rights, by abortion advocates across the world. The “rights-talk” strategy, though successful, is more fragile than it appears and has been contested by conservative and religious actors also framing anti-abortion messages in terms of human rights (rights to religious freedom, rights of the unborn child etc.) [42]. Nyanzi [43] has pointed out that framing sexual and reproductive rights as a human right can and has had important backlash effects in African contexts, especially in issues related to the situation of sexual minorities. This may explain why the actors working to expand access to safe abortion services avoid using the “rights” term, though their explanations and articulations of the topic often present it as such.

The actors working to limit access to abortion services often drew on the ‘medical’ elements of the law, such as the need of signature from medical doctors and the focus on physical and mental health in the wording of the allowed grounds for abortions. This way of reasoning resonates with a strong biomedical discourse around reproduction. Several anthropological studies of reproduction have described how presenting women’s bodies, and reproductive processes as purely biomedical phenomena has worked to paint reproduction as something biomedical and scientific and thereby outside of what is considered political [44–47]. Emphasis on the ‘medical’ aspects of the law, discursively places abortion as something governed by the secular, scientific gaze of medical practitioners, and not by a woman making reproductive choices. Interestingly, this strategy, using primarily secular and scientific arguments, is most strongly employed by religious organizations, who limited their use of religious arguments considerably. Debating sexuality and reproduction in primarily secular terms, focusing on science and data instead of morals and religion, is a strategy employed by religious organizations on national and international levels across the world and has been given the name of strategic secularism [48].

When we consider who employs the different labels, an interesting pattern emerges. On the one hand, the actors who frequently label the abortion law ‘liberal’ are the same actors who criticize the law for not ensuring access to safe abortion services for those who need it, and hence indirectly argue that the current law is too restrictive. These actors include NGOs working on sexual and reproductive health issues and other advocates working for increased access to safe and legal abortions services. On the other hand, the major religious organizations who are in clear opposition to the idea of legal abortion outside of medical emergencies, label the law ‘restrictive’. These same actors explicitly supported the inclusion of the clause in the proposed Bill of Rights in 2015 and 2016 stating that ‘the right to life begins at conception’. With this clause, they worked towards making the Termination of Pregnancy Act unconstitutional, indirectly arguing that the current law which they call ‘restrictive’ is not restrictive enough. Labeling the law as either ‘liberal’ or ‘restrictive’ thus emerges as a discursive strategy that is selectively applied depending on the context at hand.

The way a policy is imagined or perceived carries great significance and may influence how the policy actually works in a particular context [19]. Shore and Wright argue that policies can be seen as contested narratives that define the problems they are addressing in a way as to “project only one viable pathway to its resolution” [19]. The diverging characterizations of the Termination of Pregnancy Act speak to such contestations over which image of the Zambian abortion policy should prevail in society at large. Arguably, by referring to the law as either ‘liberal’ or ‘restrictive’, the actors are engaged in a struggle to define the problem of access to legal abortions in a way that makes their own view the only one possible or acceptable. Considering that knowledge about the legal framework for abortion in Zambia is poor [25], also among health professionals [24], the imagery used to depict and describe the abortion policy carries great importance. If the image of the restrictive abortion law, presenting abortion as a practice meant for exceptional cases only, predominates, it is likely that a health worker, with little knowledge about the law, will not carry out abortions on socio-economic grounds, despite what is stated in the law text. In the same way that the notion of the Christian Nation has influenced health professionals’ willingness to take part in legal abortion services, the notion of a restrictive abortion law can also shape girls’ and women’s access to safe and legal ways to terminate pregnancies.

If it is rather the imagery of a liberal abortion law, allowing abortion on broad socio-economic grounds, that predominates, it is likely that medical professionals will feel more secure and comfortable providing legal abortions, also on socio-economic grounds. However, the image of the abortion law as liberal may simultaneously move attention away from actual access to legal abortion services, as it places legality rather than access in focus. In consequence, the image of a liberal law may cause further confusion and distress among girls and women seeking abortion services. If a woman in a rural area, acts upon an impression that Zambia has a liberal abortion law and seeks legal abortion in a district hospital, she runs the risk of being turned away for multiple reasons. The professional she meets may believe the service is illegal or has chosen to make use of his/ her conscientious objection, or the facility may simply not meet the required need for signatures. Confusion about the legal status of abortion emerged as a strong theme in our interviews, and health workers, activists and policy makers emphasized how they encounter confusion about the Termination of Pregnancy Act in their work. Discourses are powerful and determine what people consider to be true [23]. When the truths established by two contradicting discourses meet, on one hand the discourse of the liberal Zambian abortion law, and on the other, the discourse of the restrictive law, confusion is likely to prevail.

### Concluding remarks

In this paper, we have argued that the Zambian Termination of Pregnancy Act is an ambiguous law that leaves much room for interpretation. Though the law in 1972 appeared to be progressive, allowing abortions on socio-economic grounds, it was the restrictive elements that were in focus in the debates preceding its enactment. These same elements were further emphasized with the declaration of Zambia as a Christian Nation, and can readily be recognized in today’s abortion debate. The paper has discussed how key stakeholders engaged in disputes over the characterizations of the Zambian abortion law selectively apply discursive strategies. Drawing upon understandings from anthropology of policies, the paper argues that the imageries used to describe the law also contribute to the shaping of girls’ and women’s access to safe and legal ways to terminate pregnancies. The law is ambiguous enough to cater both for actors who want to expand and actors who want to further restrict access to safe and legal abortion. In its ambiguity it fails to provide strong and unequivocal rights to access to the services it permits and regulates. By highlighting how the Zambian abortion policy is contested by two competing discourses, we challenge the notion of a liberal Zambian abortion law and open up for further discussions on the relationship between the way a law is described and perceived by the public, and the rights to health and services to be ensured by it.
